# Efficacy of Vitamin C Treatment to Aid the Healing of Foot Ulcers in People With Diabetes: A Randomised, Placebo‐Controlled Double‐Blind Trial: The FOOT‐C Study Protocol

**DOI:** 10.1002/jfa2.70193

**Published:** 2026-08-08

**Authors:** Michelle R. Kaminski, Eleanor Thong, Shaun Mason, Gavin Abbott, Chris Lemoh, Jennifer Wong, Paige van der Pligt, Zhong Lu, Janet Golder, Jeong Hyun Kwon, Glenn D. Wadley

**Affiliations:** ^1^ Department of Podiatry Monash Health Melbourne Australia; ^2^ School of Primary and Allied Health Care Monash University Melbourne Australia; ^3^ Discipline of Podiatry School of Allied Health Human Services and Sport La Trobe University Melbourne Australia; ^4^ Workforce, Innovation, Strategy, Education and Research (WISER) Unit Allied Health Monash Health Melbourne Australia; ^5^ Department of Diabetes & Endocrinology Monash Health Clayton Australia; ^6^ Department of Diabetes & Endocrinology The Alfred Hospital Prahran Australia; ^7^ School of Exercise and Nutrition Sciences Institute for Physical Activity and Nutrition Deakin University Geelong Australia; ^8^ Department of Medicine, Western Health Melbourne Medical School The University of Melbourne Melbourne Australia; ^9^ Department of Medicine School of Clinical Sciences at Monash Health Monash University Melbourne Australia; ^10^ Department of Nutrition and Dietetics Western Health Footscray Australia; ^11^ Department of Chemical Pathology Monash Health Pathology Monash Health Melbourne Australia

**Keywords:** clinical trial protocol, diabetic foot, foot ulcer, randomised controlled trial, vitamin C

## Abstract

**Introduction:**

Diabetes‐related foot ulceration (DFU) is a serious complication, serving as a leading cause of amputation, disability, hospitalisation and a significant contributor to healthcare costs. Deficiencies in vitamin C and other micronutrients are common in DFU. Emerging evidence suggests vitamin C supplementation may enhance wound healing; however, high‐quality randomised controlled trials are lacking. Therefore, the aim of this study is to investigate the efficacy of oral vitamin C supplementation (1 g/day) for 8 weeks on wound healing in people with DFU.

**Methods:**

This is a multi‐site, parallel group, randomised, placebo‐controlled, double‐blind trial. Eighty adults with DFU will be recruited and randomly assigned to either placebo or vitamin C supplementation (500 mg twice daily). Ulcer measurements will be obtained at baseline, 2, 4, 6 and 8 weeks with all other measures collected at baseline and 8 weeks. The primary outcome measure is the percentage reduction in ulcer size (ulcer volume in mm^3^) at 8 weeks, compared to baseline. Secondary outcomes are changes in plasma vitamin C, time to 50% ulcer healing, time to complete ulcer healing, rate of healing of ulcers and adverse events. Exploratory outcomes include changes to glycaemic status, blood lipids, blood pressure, peripheral arterial perfusion, mental health and well‐being, scurvy signs and symptoms and oxidative stress.

**Discussion:**

The findings from this research will provide high‐quality evidence on the efficacy of vitamin C for wound healing in people with DFU and help to inform clinical practice regarding vitamin C supplementation alongside current standard care to aid in the healing of DFUs.

**Trial Registration:**

Australian and New Zealand Clinical Trials Registry (ACTRN12624000675527). Universal Trial Number (UTN) U1111‐1308‐1050

## 1 Introduction

Diabetes‐related foot disease (DFD) encompasses peripheral neuropathy, peripheral artery disease, ulceration, infection, gangrene, amputation and/or Charcot neuro‐osteoarthropathy [1]. DFD affects ∼200 million people worldwide and accounts for 2% of the global disease burden [2, 3], ranking it the 13th largest cause of the world's total disease burden [2, 3, 4, 5]. Diabetes‐related foot ulceration (DFU) is a common and serious complication of diabetes with a global prevalence of 6.3% [5]. Given that around 50% of DFUs will become infected [6, 7, 8] and 20% will result in lower limb amputation [7, 9], this makes DFU a leading cause of disability and hospitalisation [10, 11, 12, 13]. The condition significantly impacts a person's quality of life [2, 4, 14, 15] and places a substantial financial burden on healthcare systems and society [12, 16, 17, 18]. Therefore, new evidence‐based therapies to complement current standard care are urgently needed.

Vitamin C is a co‐factor required for collagen formation and thus plays an essential role in wound healing [19]. Vitamin C is also a potent antioxidant and supports various cellular functions of the immune system, both of which are important for healing and the prevention and management of infection, respectively [19]. In high‐income countries, the intake of vitamin C is generally thought to be more than adequate, and low or deficient levels of vitamin C are assumed to be rare [20]. However, recent evidence suggests there is a high prevalence of vitamin C deficiency in various adult hospitalised patient populations, some of which are likely to include people at risk of DFUs [20]. Furthermore, we [21] and others [22, 23] have reported that 50%–60% of patients with foot ulcers attending high‐risk foot services located in several major Australian cities are deplete or deficient in vitamin C.

Despite the strong association between low vitamin C levels and high levels of amputation in people with DFU [21, 23], there is a lack of high‐quality randomised controlled trials (RCTs) to support vitamin C supplementation for promoting the healing of DFUs. Indeed, a recent systematic review on the role of vitamin C supplementation in the management of DFUs found only three published human trials with the main conclusion being the need for additional human clinical trials on vitamin C supplementation in people with a DFU [24]. The most convincing evidence to date is by Gunton et al. [22] who found vitamin C supplementation improved foot ulcer healing by ∼50% in a cohort of 16 patients, of which half in the cohort had diabetes. Although this data is promising, it is limited by the small sample size.

The lack of high‐quality evidence in the field highlights the need for high‐quality RCTs in people with diabetes. Therefore, the aim of this study is to investigate the efficacy of oral vitamin C supplementation (1 g/day) for 8 weeks on wound healing in people with DFU. It is hypothesised that vitamin C supplementation (1 g/day) for 8 weeks will improve the percentage of ulcer healing in people with DFU.

## 2 Materials and Methods

### 2.1 Study Design

The FOOT ulcer treatment with vitamin C (FOOT‐C) study is a multi‐site, parallel group, randomised, placebo‐controlled, double‐blind trial. The study has been registered with the Australian and New Zealand Clinical Trials Registry (ACTRN12624000675527). This study protocol has been developed using the SPIRIT guidelines [25]. The associated checklist from the 2022 extension of the SPIRIT 2013 statement [26] is included as a supplemental file and the participant timeline is shown in Figure 1.

FIGURE 1SPIRIT diagram of enrolment, interventions, assessments and outcome measures in the FOOT‐C study. ALP: Alkaline phosphatase, ALT: Alanine aminotransferase, CES‐D: Centre for Epidemiologic Studies Depression, DDS17: Diabetes Distress Scale‐17, eGFR: estimated glomerular filtration rate, GGT: Gamma glutamyl transpeptidase, HbA1c: Haemoglobin A1c, SINBAD: Site, Ischaemia, Neuropathy, Bacterial Infection, and Depth, WIfI: Wound, Ischaemia, foot Infection.

**Figure:**
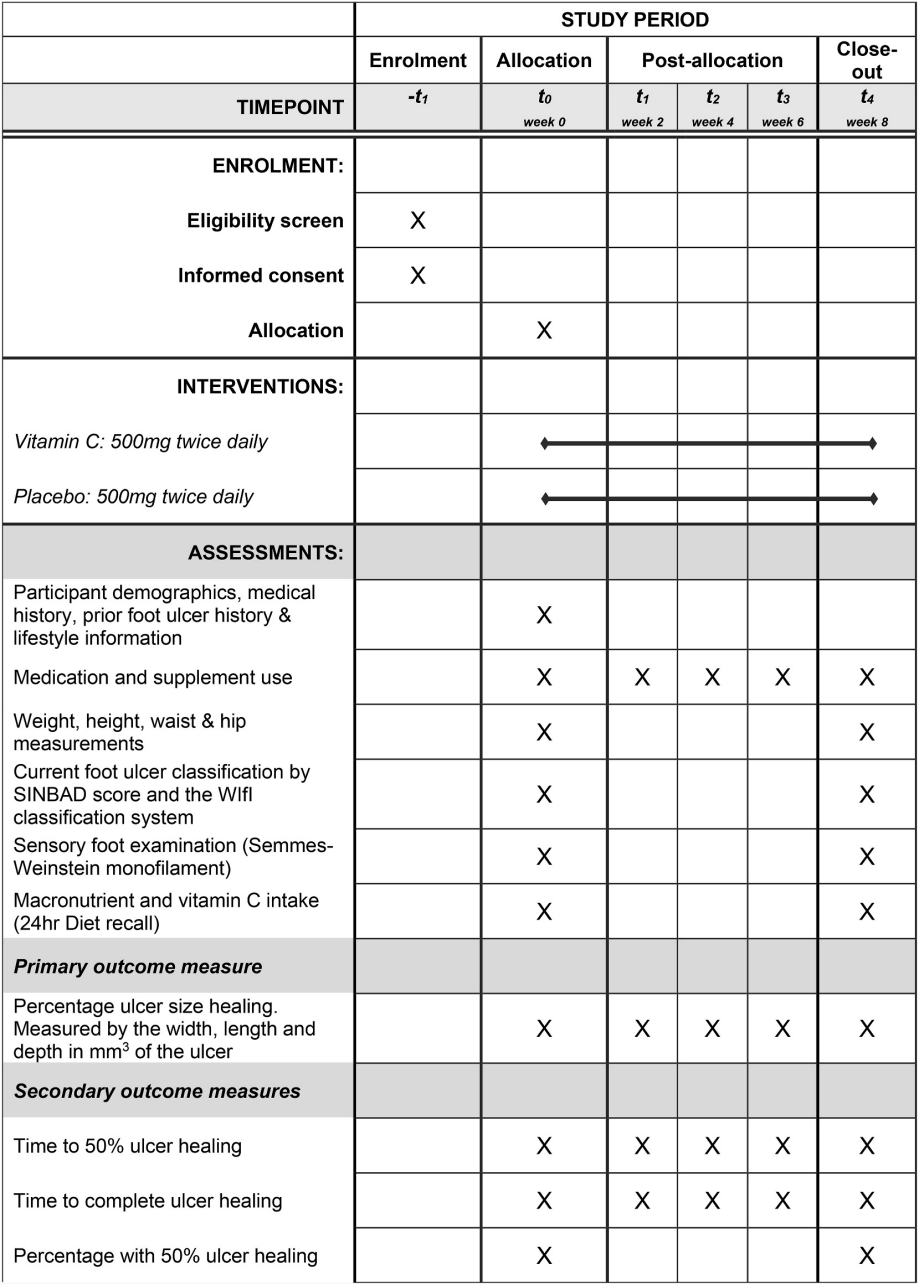

**Figure:**
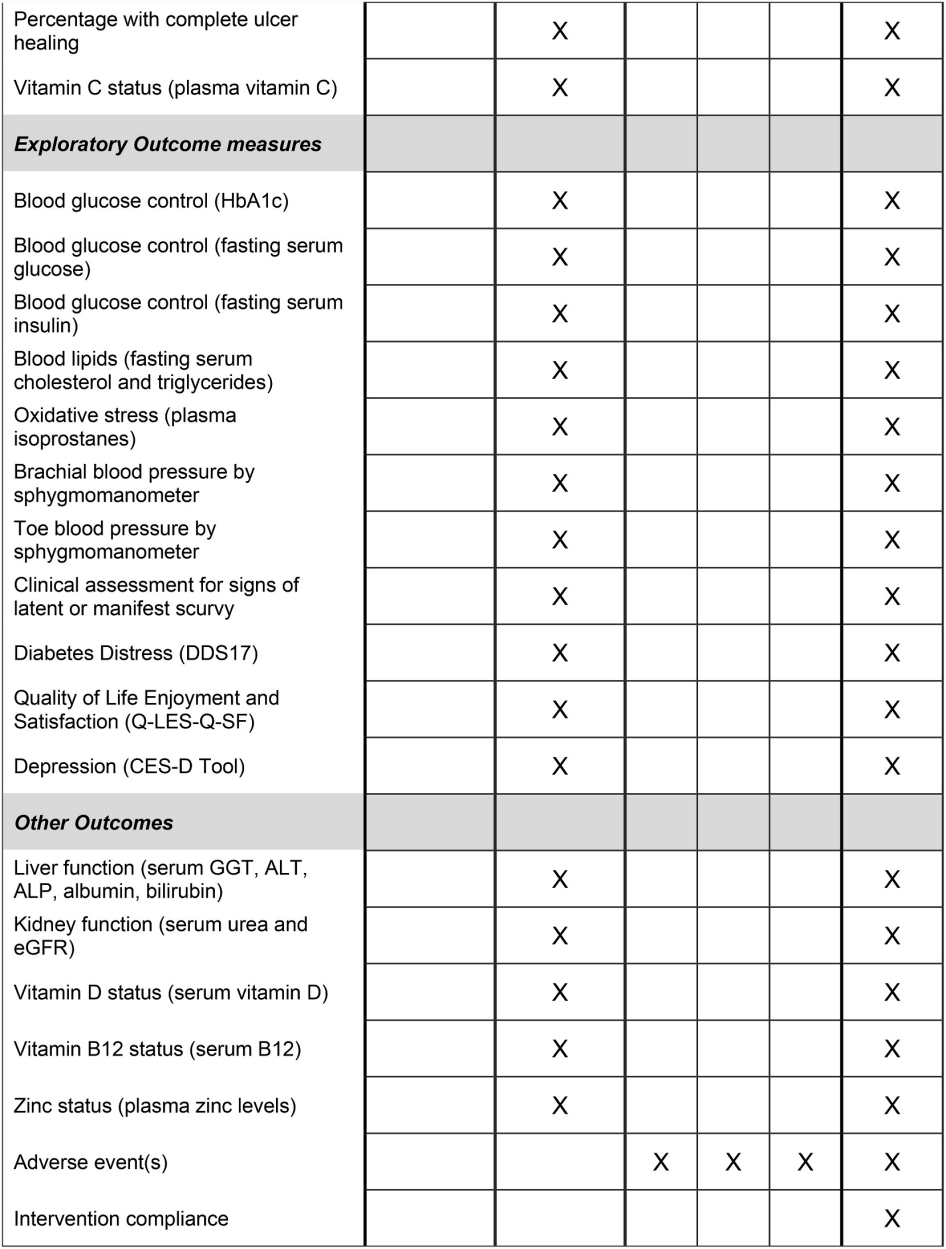

### 2.2 Intervention

Adults with a DFU will be recruited and undergo placebo or vitamin C supplementation in a double‐blind, randomised manner. The treatments will consist of oral ingestion of either vitamin C (500 mg ascorbic acid per capsule, two capsules per day; so, a total of 1 g/day) or placebo (microcrystalline cellulose capsule, two capsules per day) for 8 weeks. A placebo group as the comparator is considered the most rigorous test of treatment efficacy for evaluating a medical therapy in a double‐blind RCT [27]. Participants will be instructed to take one capsule in the morning and one capsule in the evening alongside meals. All outcome measures will be conducted at the beginning (baseline) and then at 8 weeks of treatment. Outcome measures for wound healing will also be recorded at fortnightly visits to the clinic during the 8‐week treatment period (Figure 1). The trial will conclude for each participant at 8 weeks or beforehand if the patient undergoes amputation or withdraws consent.

All participants will continue to receive usual standard of care throughout the trial. Standard care was defined according to the IWGDF Practical Guidelines (2023 update) [28] and comprised core evidence‐based prevention and management practices for DFD. This included routine risk‐based foot screening using structured assessments for neuropathy, peripheral artery disease, deformity, callus, pre‐ulcerative lesions, and any history of ulceration or amputation. Participants also received education on daily foot inspection, protective footwear use, foot hygiene, and prompt escalation for any foot concerns. Standard care additionally involved appropriate offloading and therapeutic footwear to reduce plantar pressure, as well as timely ulcer classification and management using best practice wound care and coordinated evaluation of infection or peripheral artery disease within a multidisciplinary model.

### 2.3 Ethics

Ethics approval has been obtained from Monash Health Human Research Ethics Committee (RES‐24‐0000‐298A) and the approved project has been noted and accepted by the Deakin University Human Research Ethics Committee (2024‐240). Participants will be informed about the nature of the research, first through discussions with the researchers explaining the study and then through the participant information and consent form. The participant must then provide voluntary written consent to take part in the study. Only after a participant provides informed consent for the study and additional consent for blood collection and storage will they be assessed for eligibility.

### 2.4 Participants

Eighty participants will be recruited. The inclusion criteria are adult patients (≥ 18 years old) with either type 1 or type 2 diabetes who present to the high‐risk foot service at multiple sites of Monash Health, Melbourne, Australia, with a foot ulcer. The exclusion criteria are: (i) inability to provide informed consent; (ii) already taking a vitamin C supplement; (iii) already proceeding to amputation following first visit; (iv) haemochromatosis; (v) people on dialysis or proceeding to dialysis following first visit and (vi) injuries arising from prolonged hospitalisation or immobility (i.e., pressure injuries) as these are not typically considered to have the same aetiology as a DFU.

### 2.5 Recruitment

Prospective participants will initially be identified by the medical/podiatry team within the high‐risk foot service at Monash Health, which comprises two study sites: Monash Medical Centre and Dandenong Hospital. Prospective participants will then be approached by the research assistant during the patients' scheduled appointment/s at the high‐risk foot service. The research assistant will provide participants with verbal and written information about the study if they indicate that they are interested in participating. The participant will be encouraged to contact the research team if they are interested in participating or alternatively, they will be offered a follow‐up telephone call.

### 2.6 Randomisation, Concealment and Blinding

To ensure a similar distribution of wound severity between treatments at baseline, randomisation will be stratified by study site and by ulcer size (≤ 1000 mm^3^ or > 1000 mm^3^) with random block sizes of two and four in a 1:1 ratio. Participants will be centrally randomised to treatment by an independent researcher (known as the randomisation personnel) using a computer‐generated random treatment allocation sequence uploaded onto a secure web platform (REDCap—Research Electronic Data Capture, Vanderbilt University, USA) [29, 30] hosted at Deakin University. The biostatistician on the study team will generate and upload the allocation sequence into REDCap but will have no role in recruitment or data collection. Allocation concealment is facilitated in REDCap by having different user rights and none of the investigators involved in recruitment and data collection or the randomisation personnel will have user rights to access the uploaded allocation sequence. Treatment (placebo and vitamin C) capsules will be in numbered plastic bottles that are otherwise identical. The randomisation personnel will assign numbers to the placebo and vitamin C bottles and only the randomisation personnel will know which treatment is in the numbered bottles. All baseline assessments will be completed prior to treatment allocation.

The randomisation code for an individual participant may only be unblinded in emergency situations, where the Principal Investigator (PI) decides a participant cannot be adequately treated without knowing the identity of their treatment allocation. To break the code, the PI will instruct the randomisation personnel to provide this information directly to the participant's physician/medical team. The time, date, participant code and reason for unblinding will be documented and all members of the research team will remain blinded to treatment during the study.

### 2.7 Assessment Methods

#### 2.7.1 Assessment of Parameters in Blood

At baseline and at 8 weeks completion, overnight fasted venous blood samples (approximately 29 mLs) will be taken by qualified blood collection staff at Monash Health Pathology into four tubes containing appropriate preservatives for different tests. All the serum and plasma samples will be stored at −80 degrees Celsius until batch analysis at the completion of the study. Vitamin C, oxalate and isoprostane levels will be analysed at Deakin University while all others at Monash Health Pathology.

The collected samples will be analysed by Monash Health Pathology for the measurement of glycaemic control, lipids, liver and kidney functions and micronutrients. HbA1c for glycaemic control will be measured by capillary electrophoresis in fresh ethylenediaminetetraacetic acid (EDTA) whole blood in real time at Monash Health Pathology. Glycaemic control will also be assessed by fasting serum glucose from the glucose oxidase assay and insulin by immunoassay. Blood lipid profile will be assessed by fasting serum cholesterol (total cholesterol and HDL‐cholesterol by direct measurement, and LDL‐cholesterol by calculation using the widely used Friedewald equation [31]) and triglycerides. Liver (serum gamma glutamyl transpeptidase, alkaline phosphatase, alanine aminotransferase, albumin and bilirubin) and kidney (serum urea and eGFR) function tests, lipids, vitamin B12 and vitamin D are measured in serum, while zinc in plasma from lithium heparin trace metal tubes using the Beckman Coulter automatic chemistry or immunoassay analysers.

Aliquots of EDTA with dithiothreitol added (EDTA + DTT) plasma [32] will be frozen and stored at Deakin University at −80 degrees Celsius until the conclusion of the study where it will be analysed for vitamin C [33, 34, 35], oxalate [36] and isoprostane levels [33]. Vitamin C will be measured by high performance liquid chromatography with UV detection [33, 34, 35], oxalate with liquid chromatography–tandem mass spectrometry [36] and isoprostanes (for the measurement of oxidative stress) levels by gas chromatography‐mass spectrometry [33].

#### 2.7.2 Collection of Health Information From Each Participant

At baseline, information will be collected in person by the research team regarding demographics, smoking status, alcohol intake, prior foot ulceration and medical history.

The following data will also be collected at baseline and then immediately after the 8‐week treatment (Figure 1): Height, weight, waist and hip circumference; evidence of any endovascular procedures, absence or presence of peripheral neuropathy (assessed via 10g Semmes‐Weinstein monofilament test) [37], current medication and supplement use, brachial blood pressure and peripheral arterial perfusion. The participant will be examined for scurvy signs and symptoms. Measures of mental health and well‐being and dietary intake will be obtained in person by members of the research team using validated short surveys.

#### 2.7.3 Ulcer Assessment

Ulcer assessments and measurements will be collected in person by two qualified and experienced (6 and 15 years) Podiatrists at Monash Health who are members of the research team. The number of current ulcers will be recorded. If the participant has more than one ulcer, the largest ulcer will be chosen as the index ulcer [38]. Thereafter, data will only be collected on the index ulcer for this study. Ulcers will then be classified via the Site, Ischaemia, Neuropathy, Bacterial Infection, and Depth (SINBAD) score [39], the WIfI (Wound, Ischaemia, foot Infection) classification [39] and aetiology (neuropathic, ischaemic or neuroischaemic). Ulcer duration and whether the wound is probing to bone, tissue microbiological culture results and any treatment as part of standard care, such as treatment for infection, revascularisation or pressure offloading will be recorded [38]. Amputation outcomes will also be collected, and participants undergoing amputations will be considered not to have healed ulcers.

Ulcer size will be determined by measuring the ulcer length and width with a paper ruler and the depth with a metal probe to calculate the volume in mm^3^. This approach is widely used in a clinical setting [40, 41, 42]. Bedside excisional debridement of nonviable tissue will be conducted prior to wound measurements [28]. Ulcer length will be recorded as the maximum diameter of the ulcer, while ulcer width will be taken as the perpendicular diameter to ulcer length. The ulcer area will be calculated using the ellipse formula [41] and ulcer volume calculated as ulcer area × ulcer depth. A digital image of the wound will also be captured. Interobserver reliability of standard ruler measurements has previously been reported to be excellent for both estimated area (Intraclass correlation coefficients (ICC) 0.98, 95% CI 0.97–0.98) and depth using a probe (ICC 0.93, 95% CI 0.90–0.95) [41]. Also, at the beginning of the study, both Podiatrists underwent the same training according to standard operating procedures for wound measurement that we developed for the trial based on the IWGDF Practical Guidelines (2023 update) [28] and the reliability of wound measurement study by Lasschuit et al. [41].

#### 2.7.4 Brachial Blood Pressure

Brachial blood pressure will be assessed in a supine rested position using an automated sphygmomanometer with the average of three measurements taken 3 min apart. Both arms will be measured and the value from the highest arm will be taken as brachial blood pressure [43].

#### 2.7.5 Peripheral Arterial Perfusion

Small vessel arterial perfusion and presence of peripheral artery disease will be assessed by measuring systolic toe pressures using the photoplethysmography technique [44] and calculating the toe‐brachial pressure index [45].

#### 2.7.6 Scurvy Signs and Symptoms

Symptoms and signs of latent and manifest scurvy will be assessed by a clinical assessment involving an in‐person interview asking participants if they are experiencing scurvy symptoms followed by an examination of the participants' scalp, mouth and skin of their trunk, arms and legs, for signs of scurvy [46, 47].

#### 2.7.7 Mental Health and Well‐Being

Given the high prevalence and impact on health [48], mental health outcomes will be recorded using the Diabetes Distress Screening Scale (DDS17), Centre for Epidemiologic Studies Depression (CES‐D) Tool and the Quality of Life Enjoyment and Satisfaction Questionnaire Short Form (Q‐LES‐Q‐SF) as described [49, 50, 51].

#### 2.7.8 Dietary Intake

A 24‐h diet recall will be used to assess the macronutrient and vitamin C intake using the Easy Diet Diary platform created by Xyris Software and linked to the FoodWorks database [33, 52] containing the nutrient content of > 40,000 different foods available in Australia.

### 2.8 Outcome Measures

#### 2.8.1 Primary Outcome

The primary outcome measure is the percentage reduction in ulcer size (ulcer volume in mm^3^) at 8 weeks, compared to baseline. We based our treatment length on the intervention published by Gunton et al. who found in an RCT of 16 participants that percentage ulcer healing at 8 weeks (percentage reduction in ulcer size) was significantly better in people following vitamin C treatment at median 100% compared with −14% following placebo [22]. The minimal clinically important difference is considered a 50% improvement in ulcer size after 4 weeks of treatment and is a robust predictor of complete healing in DFUs [53] and is also considered a large effect size [22, 54]. The ulcer will be considered 100% healed when the epithelium is completely intact. Outcome measures and time points are shown in Figure 1.

#### 2.8.2 Secondary Outcomes

##### 2.8.2.1 Wound Healing

The following secondary outcomes measures for wound healing are based on the measurement of ulcer volume and will be assessed by measuring at baseline and then every 2 weeks during the 8‐week treatment: (i) Time to 50% ulcer healing; (ii) Time to complete ulcer healing; (iii) Percentage of participants with 50% ulcer healing at 8 weeks and (iv) Percentage with complete ulcer healing at 8 weeks; with the ulcer being considered 100% healed when the epithelium is completely intact.

##### 2.8.2.2 Vitamin C Status

Vitamin C status will be assessed by measuring fasting plasma vitamin C at baseline and immediately after the 8‐week treatment. Although vitamin C depletion/hypovitaminosis is not universally defined, we have defined the following criteria for plasma vitamin C status (μmol/L): Normal ≥ 23; Depletion 22.9–11.4; Deficient < 11.4 [19, 46].

#### 2.8.3 Exploratory Outcomes

All exploratory outcomes will be measured at baseline and then immediately after the 8‐week treatment for glycaemic control, brachial blood pressure, peripheral arterial perfusion, scurvy signs and symptoms, blood lipid profile, oxidative stress and mental health and well‐being.

### 2.9 Adverse Events

Adverse events will be assessed and documented at every fortnightly visit for each participant by asking them whether they have experienced any unusual symptoms or medical problems since their previous study visit. As much detail as possible regarding the nature, severity and duration of the adverse event will be recorded. Bloating and diarrhoea are uncommon side effects of vitamin C supplementation and thus considered adverse reactions rather than adverse events, but they will still be documented and reported in any publication. Serious adverse events will be defined as events that result in death, are life‐threatening, require hospitalisation or the prolongation of an existing hospitalisation, or lead to persistent or significant disability or incapacity [55]. Participants will also be provided with 24‐h contact information for the study team and are encouraged to report any issues that may require medical attention.

### 2.10 Concomitant Care and Interventions

At every fortnightly visit following baseline, study staff will inquire about and record any changes to participants' medication/s, supplement usage or ulcer treatment. Participants will also be asked if they have seen a doctor or been hospitalised for any other medical reasons since their last visit.

### 2.11 Intervention Adherence

At every fortnightly visit following baseline, study staff will inquire about and record participants' adherence with the study treatment. Remaining capsules will be counted by returned bottles at the end of the 8‐week treatment period. Capsule compliance will be calculated as: (number capsules consumed divided by total number of capsules for 100% compliance) × 100%.

### 2.12 Handling of Withdrawals

When scenarios of participant withdrawal occur for reasons such as voluntary withdrawal, medical withdrawal (including undergoing amputation), protocol violation and/or adverse events, the participant's data will be included in an ‘intention to treat’ analysis (as detailed below in 2.15). No data substitution will be applied for reporting of adverse events or intervention adherence.

### 2.13 Data Management

Identifiable and re‐identifiable data will be stored separately and securely at Monash Health and at Deakin University. Hard copy data will be stored in a locked filing system at each of the study sites. All de‐identified research data will be entered directly into REDCap [30] and will be stored on a secure password protected server on the Deakin University Research Data Store. Access to all data will be restricted to members of the research team. Data will be stored for a period of 15 years after final publication of research outcomes. The collected blood samples will be labelled with codes and either measured immediately or frozen in aliquots and stored at −80 degrees Celsius at Monash Health and Deakin University for batch analysis at study completion. Following analysis at Monash Health, any remaining sample will be destroyed. At Deakin University, any left‐over plasma samples will be kept for up to 15 years and may be used for other purposes in the future closely aligned with the original research (micronutrients, oxidative stress biomarkers).

### 2.14 Sample Size

Sixty‐eight participants (*N* = 34 per treatment group) will allow for the detection of differences between treatments for the primary outcome measure of percentage reduction in ulcer size after 8 weeks of treatment. Since standard deviation (SD) values have not been published for the primary outcome measure, we assumed a large effect size of Cohen's *d* = 0.80 (*α* = 0.05, two‐tailed), with estimated power of 90%. The chosen effect size was based on prior studies that have either reported ulcer size healing after several weeks of vitamin C treatment or placebo [22] or the difference in ulcer size reduction for ulcers that healed compared to those that did not heal following several weeks of standard care [54]. Therefore, to account for an expected 15% participant withdrawal, we will recruit 80 participants to ensure sufficient power for our primary outcome measure. This sample size will also provide 90% power to detect a minimum 3.5 hazard ratio for time to 50% ulcer healing, assuming censoring of 70% in the control group [22].

Estimated power is ≥ 99% for the secondary outcome measure of 50% increase in plasma vitamin C levels after 8 weeks of treatment (mean difference = 48 μmol/L, SD = 19 μmol/L) [33] (*α* = 0.05, two‐tailed). To inform sample size calculations for future RCTs, exploratory outcome measures will also be collected at baseline and at the end of the treatment with estimated power to detect clinically meaningful differences for the following measures being between 27% and 61% (HbA1c, fasting glucose, systolic blood pressure, mental health and well‐being surveys).

### 2.15 Statistical Analysis

Primary analyses will be conducted on an intention‐to‐treat basis. Treatment effects for continuous outcomes will be estimated using linear regression models, adjusted for stratification variables study site and baseline ulcer size, and where relevant also baseline level of the outcome. For time‐to‐event outcomes, time to 50% healing and time to complete healing, we will conduct survival analysis, including Kaplan–Meier curves and Cox proportional hazards models (adjusted for study site and baseline ulcer size). Multiple imputation by chained equations will be used to handle missing outcomes data (except for censored time‐to‐event data). Per‐protocol analysis will also be performed on all participants who are > 90% capsule compliant. Complete case analysis will also be conducted to examine if results were sensitive to the approach for handling missing data. Statistical analyses will be conducted using Stata/SE 17.

## 3 Discussion

The aim of this RCT is to evaluate the efficacy of oral vitamin C supplementation on wound healing in people with DFU. There is now sufficient evidence from us and others that low vitamin C status is highly prevalent in patients with DFUs [20, 21, 22, 23], and this is likely associated with sub‐optimal wound healing and higher risk of amputation [21, 23].

Our RCT has several strengths. First, to minimise the risk of bias we are using independent researchers for allocation concealment and randomisation. This ensures that both investigators and participants remain blinded during data collection and analysis. Additionally, we have tried to limit the exclusion criteria so that the study findings will likely be generalisable to other patients with DFU who attend a high‐risk foot service. Also, the outcome measures are all routinely utilised in high‐risk foot services, further enhancing the study's clinical relevance. Should the findings be positive, the intervention has strong potential for widespread implementation due to its low cost, commercial availability and minimal risk of side effects.

Our study has some limitations to be considered. The 8‐week duration may be insufficient to capture complete ulcer healing in all participants. Additionally, the study is also not likely sufficiently powered to address other questions, including if there is a threshold of plasma vitamin C concentration above which there is no benefit of supplementation for wound healing. Furthermore, it is beyond the scope of this trial to establish the efficacy of vitamin C supplementation on other clinically important outcomes such as amputation rates and mortality. However, percentage changes to wounds after just 3–4 weeks are sufficient to provide robust predictions of complete ulcer healing [53, 56]. Thus, the duration of treatment is sufficient to properly address the aims of this study and provide clinically relevant outcomes.

An important consideration in our study is the dose of vitamin C. While the 1 g/day oral dose of vitamin C is high compared to the recommended daily intake of 40 mg/day, it is a common commercially available dose and proposed as a ‘prudent’ upper level of intake by the NHMRC [19]. Regarding tolerability, no studies have reported any adverse effects with several months' treatment of 1 g/day vitamin C supplementation in humans (reviewed in [19]).

### 3.1 Trial Status and Plan for Dissemination of Findings

This study has commenced recruitment, and the first participant completed their baseline assessment in January 2025. We anticipate data collection from all participants to be completed by June 2026. We anticipate the trial will produce one high‐quality publication and we will communicate our findings at relevant scientific meetings. We are also capturing several secondary and exploratory outcome measures to provide pilot data to inform future studies and provide opportunities for future research using an approach consistent with the objectives from the Diabetes Feet Australia national strategy [57].

## Author Contributions

**Michelle R. Kaminski:** conceptualization, methodology, supervision, writing – original draft, writing – review and editing. **Eleanor Thong:** conceptualization, methodology, supervision, writing – review and editing. **Shaun Mason:** conceptualization, methodology, writing – review and editing. **Gavin Abbott:** conceptualization, methodology, writing – review and editing. **Chris Lemoh:** conceptualization, methodology, writing – review and editing. **Jennifer Wong:** conceptualization, methodology, writing – review and editing. **Paige van der Pligt:** conceptualization, methodology, writing – review and editing. **Zhong Lu:** conceptualization, methodology, writing – review and editing. **Janet Golder:** conceptualization, methodology, writing – review and editing. **Jeong Hyun Kwon:** methodology, writing – review and editing. **Glenn D. Wadley:** conceptualization, methodology, supervision, writing – original draft, writing – review and editing.

## Funding

This study was funded by a grant awarded from the Diabetes Australia Research Programme. Trial sponsor: Deakin University, Australia. Roles of trial sponsor and funder: Deakin University (Sponsor) and Diabetes Australia (Funder) have no role in study design, the collection, management, analysis and interpretation of data, the writing of the report and the decision to submit the report for publication. This work was supported by Diabetes Australia (Y24G‐WADG).

## Conflicts of Interest

The authors declare no conflicts of interest.

## Data Availability

There are currently no plans for sharing data or future use of this data.
